# Vertèbre de poisson impactée dans le larynx d’un nourrisson: à propos d’un cas inhabituel

**DOI:** 10.11604/pamj.2025.51.19.47322

**Published:** 2025-05-22

**Authors:** Aminata Diop Nakoulima, Khadidia Fall, Mame Rouba Ndiaye, Mame Diarra Mbacke, Mame Diarra Bousso Ndao, Cheikh Ahmédou Lame

**Affiliations:** 1 Département Mère Enfant, Hôpital Principal de Dakar, Dakar, Sénégal,; 2 Service ORL, Hôpital Principal de Dakar, Dakar, Sénégal,; 3 Service d´Imagerie Médicale, Hôpital Principal de Dakar, Dakar, Sénégal

**Keywords:** Corps étranger inhalé, larynx, arête de poisson, cas clinique, Foreign body aspiration, larynx, fishbone, case report

## Abstract

L'inhalation de corps étrangers (CE), accident fréquent chez l'enfant, peut mettre en jeu le pronostic vital. Différents types de corps étrangers peuvent être retrouvés, mais la localisation laryngée est rare. Nous rapportons le cas d´un nourrisson de sexe masculin, âgé de 8 mois, qui a été reçu pour difficulté respiratoire avec dysphonie et stridor. A l'admission, l'enfant présentait une dyspnée laryngée sévère. La tomodensitométrie cervicale retrouvait un corps étranger radio-opaque laryngé. L'extraction endoscopique ramenait une vertèbre de poisson impactée dans la sous-glotte. Les corps étrangers des voies respiratoires inférieures de localisation laryngée sont rares et la découverte de vertèbre de poisson exceptionnelle.

## Abstract (en)

Inhalation of foreign bodies, a common occurrence in children, can be life-threatening. Different types of foreign bodies can be found, but laryngeal localization is rare. We here report the case of an 8-month-old male infant who presented with respiratory distress, dysphonia, and stridor. On admission, the child exhibited severe laryngeal dyspnea. Cervical CT scan revealed a radiopaque foreign body in the larynx. Endoscopic extraction revealed an impacted fish vertebra in the subglottis. Foreign bodies located in the laryngeal area of the lower airways are rare, and the discovery of a fish vertebra is exceptional.

## Introduction

L'inhalation de corps étrangers (CE) est un accident fréquent en pratique pédiatrique. Elle peut mettre en jeu rapidement le pronostic vital [1-3]. La localisation laryngée est la plus rare au niveau des voies respiratoires inférieures [3]. Si la graine d'arachide constitue le CE le plus fréquemment inhalé par l'enfant [2], la vertèbre de poisson peut être exceptionnellement retrouvée dans le larynx [1]. Les auteurs rapportent une observation de vertèbre de poisson inhalée et plantée dans la sous-glotte d´un nourrisson de 8 mois.

## Patient et observation

**Information du patient:** un nourrisson de 8 mois de sexe masculin, sans antécédent pathologique, nous a été référé par un hôpital pédiatrique pour prise en charge d´une détresse respiratoire.

**Résultats cliniques:** l'interrogatoire de la mère retrouvait une notion de suffocation suivie de quintes de toux émétisante survenue brutalement au cours d'un repas. Le garçon était redevenu asymptomatique après cet épisode. Devant l'apparition secondaire d'une dyspnée, il a consulté dans un hôpital pédiatrique où une antibio-corticothérapie associée à des séances de nébulisation d´adrénaline a été instituée. Quarante-huit heures après, devant l'aggravation du tableau malgré le traitement, le patient nous a alors été référé.

A l'admission, le nourrisson était obnubilé, avec une dyspnée inspiratoire, un tirage intercostal et un balancement thoraco-abdominal. Le cri était étouffé. L'auscultation pulmonaire retrouvait des champs pulmonaires libres.

**Figure 1:**
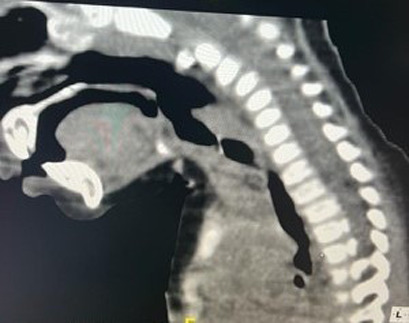
TDM cervicale en coupe sagittale montrant un corps étranger radio opaque localisé au niveau laryngé

**Figure 2:**
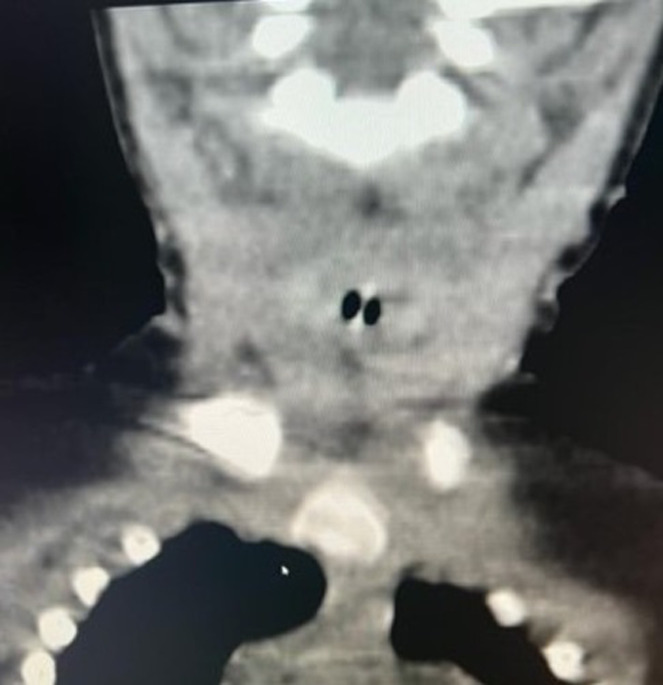
TDM cervicale en coupe coronale montrant un corps étranger radio opaque localisé au niveau laryngé

**Figure 3:**
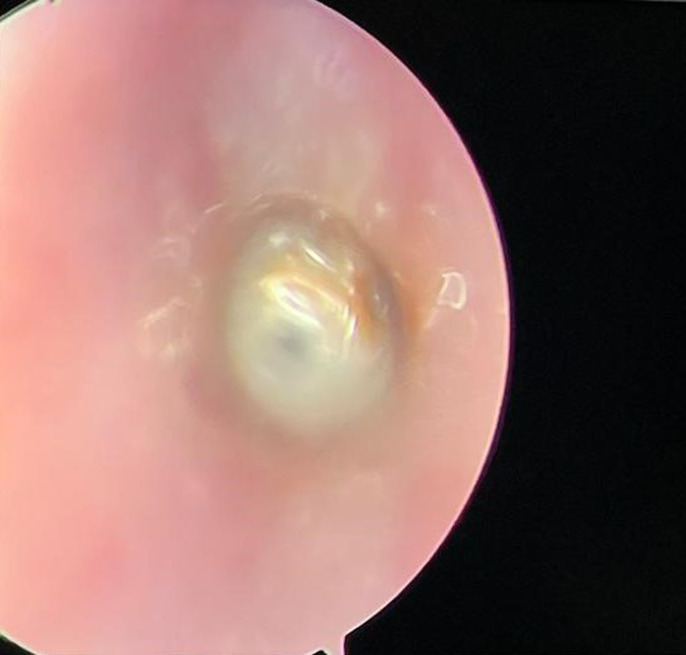
corps étranger sous-glottique recouvert de sécrétions

**Démarche diagnostique:** la tomodensitométrie cervico-thoracique, faite en urgence, mettait en évidence la présence d´un CE, radio opaque, situé en position laryngée (Figure 1, Figure 2). La laryngoscopie directe effectuée sous anesthésie générale retrouvait un corps étranger sous-glottique recouvert de sécrétions séromuqueuses (Figure 3).

**Figure 4:**
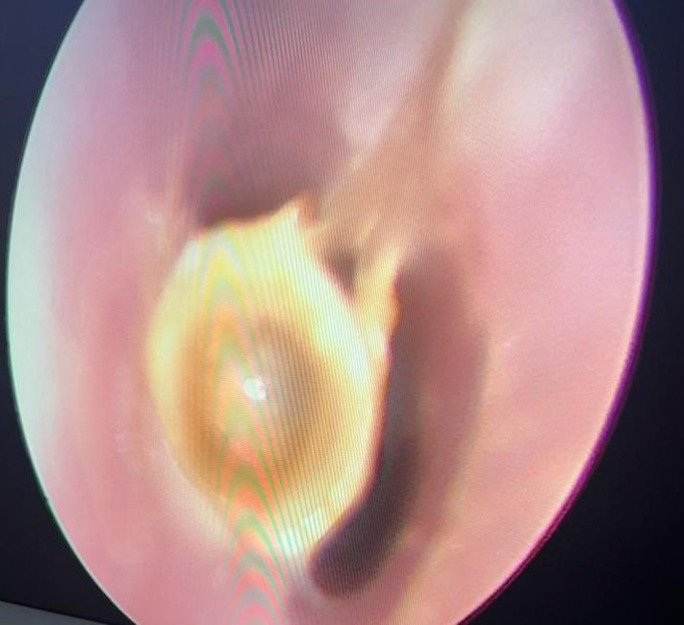
corps étranger sous-glottique

Après aspiration, le CE était visualisé, planté sous les cordes vocales (Figure 4).

**Figure 5:**
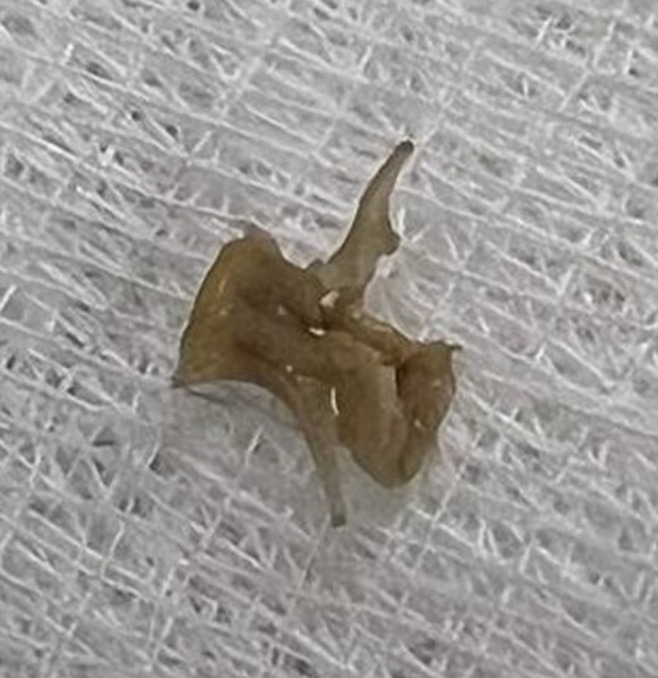
vertèbre de poisson extraite

**Intervention thérapeutique et suivi:** la préhension et l'extraction douce après désenclavement ramenaient une vertèbre de poisson (Figure 5). Les suites opératoires étaient simples.

**Consentement du patient:** le consentement pour soins a été obtenu de la mère du patient.

## Discussion

L'inhalation de CE est un accident habituellement retrouvé dans le cadre des urgences pédiatriques [1,2,4]. Sa fréquence annuelle est de 3/10000 enfants [2]. C'est un accident grave représentant 7% des décès accidentels chez l'enfant de moins de 4 ans [5], soit la 3^e^ cause de décès entre 1 et 4 ans [2]. Aux États-Unis, 150 enfants meurent chaque année par inhalation de CE [6]. Cette pathologie atteint deux fois plus les garçons que les filles [2]. La présentation clinique dépend de l'âge du patient, de la taille, de la nature et de la forme du CE [2,7]. Elle dépend aussi de la localisation et du délai de séjour du CE [2]. La localisation bronchique est la plus fréquente [3]. La graine d'arachide est le CE le plus souvent retrouvé chez l'enfant [2,5]. La vertèbre de poisson reste un corps étranger sous-glottique exceptionnel [1,8].

Le diagnostic repose sur l'existence d'un syndrome évident de pénétration, des données de l'auscultation pulmonaire et des examens radiologiques [2,4,9]. La fibroscopie laryngo-trachéo-bronchique permet de confirmer directement la présence d'un CE du volume de réserve inspiratoire (VRI). Elle est utilisée par plusieurs praticiens comme outil diagnostique avant de réaliser la bronchoscopie rigide [2].

Le traitement consiste à extraire le CE par voie endoscopique sous anesthésie générale. Il requiert une équipe expérimentée avec une bonne collaboration entre endoscopistes et anesthésistes [1,3,9] afin de réduire la morbi-mortalité liée à ce geste non anodin.

## Conclusion

Les corps étrangers des VRI constituent une urgence pédiatrique fréquente et potentiellement grave. La graine d´arachide localisée au niveau bronchique reste la forme la plus commune. La vertèbre de poisson sous-glottique est un CE laryngé inhabituel. L'imagerie et l'endoscopie sont d´un grand apport diagnostique. Le meilleur traitement reste la prévention qui passe par la sensibilisation et l´éducation des parents.
